# The ADAPT-HEAT Study: A Multi-Method Approach to Develop Recommendations for Drug Safety During Hot Weather (The CALOR List)—Study Protocol

**DOI:** 10.3390/mps9030078

**Published:** 2026-05-25

**Authors:** Maxie Bunz, Pascal Nohl-Deryk, Heike van de Sand, Katharina van Baal, Svenja Arendt, Alina Herrmann, Ingo Meyer, Adriana Poppe, Olaf Krause, Johannes Heck, Beate Sigrid Müller

**Affiliations:** 1 Institute of General Practice, Faculty of Medicine and University Hospital Cologne, University of Cologne, 50937 Cologne, Germanybeate.mueller@uk-koeln.de (B.S.M.); 2 Health Department, City of Cologne, 50667 Cologne, Germany; 3 PMV Research Group, Faculty of Medicine and University Hospital Cologne, University of Cologne, 50931 Cologne, Germany; 4 Institute for General Practice and Palliative Care, Hannover Medical School (MHH), 30625 Hannover, Germanyheck.johannes@mh-hannover.de (J.H.); 5 Institute of Global Health, Faculty of Medicine and University Hospital Heidelberg, Heidelberg University, 69120 Heidelberg, Germany

**Keywords:** hot temperature, extreme heat, patient safety, clinical protocols, potentially inappropriate medication list

## Abstract

Certain medications may adversely affect health during hot days and heatwaves by altering chronic conditions, comorbidities, fluid balance, or impairing heat adaptation. This study aims to develop evidence-based cross-sectoral recommendations for the safe administration of heat-sensitive medications, compiled into a so-called ‘CALOR’ list (calor: Latin for ‘heat’). Development of the CALOR list will follow a four-pillar process. First, a scoping review of scientific literature and best practices will identify potentially inadequate medications during heat events (heat-PIMs) and adaptation measures, resulting in a first draft. Second, an expert panel will refine this draft through a Delphi process to reach consensus on clinically relevant recommendations. Third, German statutory health insurance (SHI) claims data will be analysed to determine heat-PIMs prevalence; data from Cologne residents will additionally be linked with climate data to investigate health outcomes during heat events. Fourth, thirty health professionals (i.e., medical doctors, nurses, pharmacists) will field-test the CALOR list in summer, providing qualitative feedback on feasibility, leading to further refinement of the CALOR list. To our knowledge this study protocol presents the first study attempting to collate a comprehensive and actionable list of recommendations for drug safety management during hot days and heatwaves.

## 1. Introduction

Climate change is contributing to a rising frequency and intensity of high-temperature events (e.g., heatwaves) in Germany, as in many other countries. Since 1950, the number of hot days (≥30 °C) and tropical nights (≥20 °C) in Germany has increased by an average of 11.4 days per year [1], and this trend is projected to continue. Climate projections estimate that, by the end of the century, the proportion of hot days during the summer months (April to September) could rise from 5% to 30% of all days in urban areas of Bavaria [2]. This escalation in extreme heat events is associated with significant health risks. With a global indicator for heat-related mortality, Watts et al. (2021) estimated 20,200 heat-related deaths in 2018 among individuals aged 65 and older in Germany [3]. Only for China (62,000 deaths) and India (31,000 deaths)—each with populations around 1.4 billion—higher absolute numbers of heat-related mortality were estimated [3]. In the German federal state of Hesse, the average number of deaths during heatwaves doubled between 2000 and 2018 [4]. In its capital, Frankfurt am Main, the excess mortality rate during heat events between 2003 and 2013 reached 78%, rising to as high as 113% among those aged over 80 [5].

There is growing evidence that certain medications contribute to increased health risks during heat events. As temperatures rise, a wide range of pharmaceuticals may become hazardous through various mechanisms [6,7]. In particular, many drugs impair the body’s physiological capacity to adapt to heat [3]. For instance, anticholinergic agents (e.g., tricyclic antidepressants such as amitriptyline or antipsychotics such as olanzapine) can inhibit sweat production, impairing thermoregulation [8]. Similarly, angiotensin-converting enzyme (ACE) inhibitors (e.g., ramipril) can reduce the sensation of thirst, increasing the risk of dehydration [9]. Individuals with chronic conditions are particularly vulnerable to heat-related complications. Those with obesity, hypertension, cardiovascular disease, diabetes or pulmonary disease face significantly elevated risks for heat-induced illnesses, such as heat exhaustion or heatstroke [10]. These conditions can also affect the appropriateness of certain medications (e.g., drugs prescribed on the basis of glomerular filtration rate on days without heat stress) [6]. Finally, elevated temperatures can alter the pharmacological properties of certain medications. For example, short-acting insulins and opioid analgesics (e.g., fentanyl from transdermal patches) may exhibit accelerated influx or increased potency on hot days due to increased blood flow to the skin [11,12].

A representative survey of German general practitioners (GPs) in 2021 revealed that only 16% had adjusted medications during heat events [13]. Certain initiatives in Germany have begun to identify medications that may pose a risk during extreme heat and offer preliminary, experience-based recommendations for their adjustment [14]. For example, the Hannover Medical School has developed and published an expert guide entitled ‘*Help during heat in residential care homes*’, outlining a range of measures to mitigate the effects of heat in institutional care settings [15]. However, no standardised, evidence-based and consensus-driven recommendations for the adjustment of heat-sensitive medications are currently available to the broader healthcare profession.

The project ADAPT-HEAT (‘Adaptation of Drug Therapy During Hot Seasons’) seeks to address this critical gap by developing the ‘CALOR list’—a comprehensive, evidence-based and expert-consensus-backed tool for guiding medication adjustments in response to extreme heat. The CALOR list is intended to support clinical decision-making for the heat-sensitive adaptation of patient medication, thereby minimising side effects and heat-related hospital admissions while contributing to broader efforts to make the healthcare system more resilient to climate change.

## 2. Materials and Methods

### 2.1. Study Design

The ADAPT-HEAT study will employ a multi-methods approach to identify and synthesise existing evidence, with the goal of developing a set of practical, evidence-based recommendations for the adaptation of heat-sensitive medications. As a conceptual framework, the study is based on the methodology of the PRISCUS list, which contains potentially inappropriate medications for older adults [16]. The authors hope to establish a working definition of heat-potentially inappropriate medications (heat-PIMs) as drugs that, through one or more pharmacological mechanisms, may increase an individual’s susceptibility to heat-related illness (HRI) or whose safety and efficacy profile may be adversely affected by hot environments, and for which specific adaptation measures—such as dose adjustment, enhanced monitoring, or fluid balance management—could be warranted during heatwaves. The ADAPT-HEAT study will be conducted from January 2024 until December 2026.

### 2.2. Development of the CALOR List

**Figure 1:**
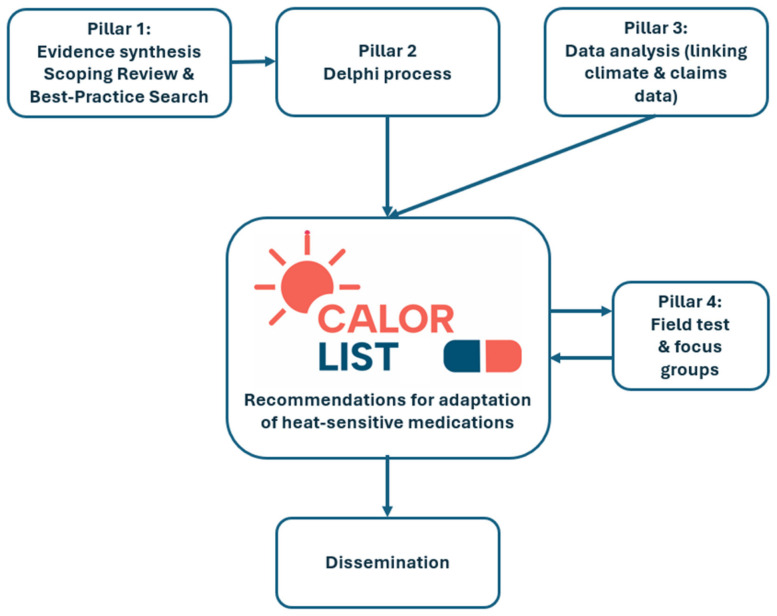
Study design.

The CALOR list will be developed through a structured, multi-pillar process comprising evidence synthesis, Delphi process and data analysis to inform recommendations for the adaptation of heat-sensitive medications (Figure 1).

### 2.3. Pillar 1: Evidence Synthesis of the International Literature and Best Practices

Initially, the development of the CALOR list will be informed by a scoping review of the scientific literature, complemented by an analysis of national and international best practice for the heat-sensitive adjustment of medication. The scoping review will aim to identify and assess studies reporting on potentially inappropriate medications during heat events and explore possible adaptation measures (e.g., dose adjustment, hydration guidelines). By summarising current knowledge, the scoping review will contribute to the development of the CALOR list. The review protocol, including the search strategy and selection criteria for evidence sources, will follow the PRISMA-ScR (Preferred Reporting Items for Systematic Reviews and Meta-Analyses—Extension for Scoping Reviews) checklist [17]. Following the PCC framework research is of interest, focusing on human population (population) and risks from medication during heat, including hot weather or hot season (concept), while helping to identify adaptation measures reducing risks from drugs during heat (context). The search will include evidence from PubMed, Web of Science, and Google Scholar. Publications will be included if written in English or German. Publications describing case reports, protocols, comments, editorials, letters, posters, preprints will be excluded. Results will also be excluded if their focus are veterinary populations, chemical studies, do not report on drugs or do not assess heat sensitivity. Further details on the search strategy can be found at https://osf.io/2abvu/overview (accessed on 13 April 2026) [18].

In the review of national and international best practice models, particular attention will be given to Spain, Italy and France—European countries with demographic profiles comparable to Germany, but with greater practical experience in managing public health during periods of extreme heat. For example, the Spanish Society of Family Medicine has already issued guidance on the protection of older adults during heat events [19].

The findings from this comprehensive evidence synthesis will inform the development of the first draft of the CALOR list by the study team.

### 2.4. Pillar 2: Delphi Process

Approximately 30 interdisciplinary experts will participate in a structured Delphi process [20] to achieve expert consensus on the practicality and clinical relevance of the preliminary recommendations for the adjustment of heat-sensitive medication identified in pillar 1.

The Delphi panel will include stakeholders and individual healthcare professionals from across Germany with recognised expertise in drug prescription, pharmacology and/or the health effects of heat. The panel will be intentionally designed to represent a broad spectrum of perspectives, including professionals from inpatient and outpatient care settings, as well as representatives of relevant professional medical and pharmaceutical societies and associations. Experts will be recruited through professional and personal networks, snowball sampling methods and targeted online research, with invitations sent via email. Participation will be voluntary, and experts who complete all rounds of the Delphi process will receive monetary compensation.

The Delphi process will be conducted as an online survey using the SoSciSurvey platform [21], and will involve two to three iterative rounds, depending on the level of agreement to the recommendations. The survey will be administered in German. In the first round, participants will be presented with the medications and associated adaptation measures identified in pillar 1. Experts will be asked to rate each adaptation recommendation for the heat-PIMs using a 4-point Likert scale (strongly agree, somewhat agree, somewhat disagree, disagree) across two dimensions: (a) clinical relevance and (b) practicability/feasibility. Experts will have the opportunity to provide free-text comments to explain their ratings or offer additional insights and suggestions. Group consensus will be reached when at least 75% of the experts agree on both criteria (relevance and practicability/feasibility) for a given recommendation [22]. The results will be evaluated using frequency analyses conducted using the SPSS software (version 29.0). Adaptation measures that do not reach consensus will be revised on the basis of the free-text comments and reintroduced in the second and, if necessary, third survey round. Each Delphi round will be administered via email, remain open for approximately 3 to 4 weeks and include reminder notifications to encourage participation.

At the conclusion of all Delphi survey rounds, the project team will consolidate all adaptation measures for heat-PIMs that achieved consensus. The CALOR list will be revised accordingly.

### 2.5. Pillar 3: Analysis of Statutory Health Insurance (SHI) Claims Data and Linked Climate Data

In this pillar, SHI claims data will be analysed to assess the national prescription prevalence of the identified heat-PIMs and to link it with heat data. The first part of the analysis will utilise claims data from BARMER, one of Germany’s largest SHI providers. This dataset comprises anonymised healthcare data from approximately 8.7 million insured individuals across Germany. The second part of the analysis will involve a linked dataset combining SHI claims data and climate data for the city of Cologne, drawing on the CoRe-Net database (CoRe-Dat) of claims data for approximately 500,000 Cologne residents over the period 2013 to 2022.

For the heat-PIMs identified in pillar 1, an analysis will be conducted using BARMER data to assess prescription frequency and related aspects (e.g., frequent co-medications, average prescription quantities) among all SHI-insured patients in the dataset. Patient characteristics—including morbidity, age and gender—will also be examined. Data will cover the period 2019 to 2023, enabling detailed temporal analyses of drug prescriptions. Additionally, both outpatient and inpatient diagnoses, as well as demographic information, will be incorporated.

Initial drug epidemiology analyses will be based solely on SHI claims data to generate key figures regarding the overall target population. Interim results (particularly those concerning prescription frequency) will be used to revise the CALOR list with regard to drug prioritisation.

Subsequently, CoRe-Dat data will be linked with weather and climate data from the Deutscher Wetterdienst—German Weather Service (DWD) and analysed analogously to the initial phase. This will provide historical metrics for the period 2013 to 2022 for patients with actual heat exposure. Unlike the BARMER dataset, the CoRe-Dat dataset includes more granular patient data, such as full residential postcode. This specificity will allow for the precise linkage of patient data with weather and climate variables at local and temporal levels.

Heat periods will be assessed using a hierarchical framework comprising (1) official heat warning information by the DWD, (2) station-based metrological observations, and (3) spatially resolved modelled estimates. The official heat warnings are based on perceived temperature (PT) by a temperature over 32 °C with only a slight drop in temperature at night [23]. The temperature thresholds for (2) and (3) are set to ≥32 °C (associated with moderate heat stress) and ≥38 °C (associated with extreme heat stress) [24]. A day is classified as heat-exposed if the maximum hourly air temperature recorded between 12:00 and 16:00 local time reaches or exceeds the temperature thresholds. The afternoon exposure window is chosen to capture the period of the highest daytime thermal load. To account for a potential delay in the effect of heat on health outcomes, two lag structures will be evaluated. The first model estimates immediate, same-day effects only (lag 0), while the second model additionally captures short-term delayed effects over a window of up to three days (lag 0–3).

In the third phase of the analysis, changes during heat periods will be examined through a specific use case (i.e., a frequently occurring medical case constellation) for the city of Cologne. This use case will be selected on the basis of the findings from pillar 1, focusing on the most relevant medication–disease combinations in the German healthcare context. To identify changes during and after heat events, CoRe-Dat data will again be used. The defined population for the use case will be compared before and during heat events, with the number of hospitalisations and mortality serving as the primary endpoints [25].

For statistical analyses, a time-stratified case-crossover design will be applied. In this approach, each participant serves as their own control, which inherently accounts for all time-invariant confounders, such as sex. This method has been widely used in public health research examining the relationship between temperature or heat exposure and health outcomes, including studies on mortality [26], emergency department visits [27], and heat-related illness [28] across various geographic settings. Given the large number of planned analyses across climate data sources, heat intensity thresholds, and lag structures, the False Discovery Rate (FDR) will be controlled using the Benjamini–Hochberg procedure to account for multiplicity [29].

These analyses will allow examination of whether patient-related factors (e.g., age, comorbidities) or specific medications modify the association between heat and health outcomes, thereby informing the refinement of recommendations for high-risk patients in the CALOR list.

Data analysis for pillar 3 is ongoing in 2026 and results are expected at the end of 2026.

### 2.6. Pillar 4: Field Test of the CALOR List

The refined version of the CALOR list used for the field test will include the names of the identified heat-PIMs alongside recommendations for their adaptation. To test the list’s applicability in clinical practice, an information letter outlining the background and importance of heat-sensitive medication adjustment will be distributed—together with the refined version of the CALOR list—to approximately 30 healthcare providers in total, including GPs, healthcare assistants, hospital physicians, nurses, pharmacists and pharmaceutical technicians. As this study follows a qualitative methodological approach, a statistical calculation of sample size is not undertaken; rather, purposive sampling is applied. Recruitment will be conducted as a maximum-variation-sampling by the Institute of General Practice at the University of Cologne. Participating healthcare providers will implement the CALOR list within their respective setting (e.g., ambulatory practices, hospitals, pharmacies) over a 3-month period during summer (June through August). Their experiences will be evaluated through online focus groups comprising four to five participants each, focused on the overall usability of the list [30]. This participatory approach will facilitate both the gathering of experiential data as well as the exchange of ideas regarding challenges and potential improvements to the CALOR list to support implementation. A semi-structured interview guide will be employed to ensure comprehensive coverage of all relevant aspects. Focus group discussions will be transcribed verbatim and analysed using MAXQDA software, applying thematic analysis according to Brown and Clarke [31]. Both deductive and inductive approaches will be used, with deductive categories informed by the implementation framework of Chaudoir et al. [32]. Based on the findings from the focus groups, the CALOR list will be revised into a final version.

Recruitment for pillar 4 started on 31 March 2025 and was completed on the 7 July 2025. Data analysis is ongoing and results are expected at the end of 2026.

## 3. Discussion

The CALOR study employs a robust four-pillar methodology that integrates evidence synthesis, expert consensus, real-world claims data analysis, and field testing to produce clinically actionable recommendations for drug safety during hot weather.

Despite its methodological strengths, the ADAPT-HEAT study is subject to several limitations that should be considered. The Delphi panel, while intentionally interdisciplinary, will be limited to experts from German healthcare context, because the list is primarily intended to work within the context. This may constrain the generalisability of the resulting recommendations to other healthcare systems and climatic regions. The analysis of SHI claims data, introduces inherent limitations common to administrative data, including potential miscoding, incomplete capture of over-the-counter medications, and the absence of clinically nuanced patient information such as functional status or detailed comorbidity profiles. The combination of claims and weather data restricted to Cologne limits generalisability beyond Cologne, particularly to rural settings and other climatic regions. Further, the field test, conducted with a small sample over a single three-month summer period, may not fully capture the variability of implementation challenges across different regional climates, care settings, or patient populations. Patient involvement is ensured through the involvement of patients in the advisory group participating in bi-annual meetings. Furthermore, patients will be involved in designing patient information materials based on the CALOR-list, as outlined below.

### Dissemination Plans

The CALOR list will be made openly available to health professionals in a digital format. To ensure broad reach and comprehension, the key recommendations will be translated into various formats, using clear, accessible and lay-friendly language. Print materials (e.g., information letter, brochure, poster) will be developed for the target group of (elderly) patients. To further extend outreach, key messages and recommendations will be communicated through one or more short videos aimed at patients and their relatives, disseminated via social media and other digital platforms. By offering both print and digital resources in lay language, the initiative will facilitate low-threshold access to essential information, particularly for individuals engaging with healthcare services on only an irregular basis. Importantly, these patient-facing materials will be developed through a participatory process, incorporating feedback from 10 patients to ensure clarity and relevance.

To facilitate the long-term integration of the CALOR list into clinical practice, a range of materials will be developed to embed its recommendations within medical education and training.

(1) Medical education: A presentation, accompanied by multiple-choice questions, will be designed for use in lectures across various disciplines (e.g., general medicine, internal medicine, pharmacology, clinical pharmacology).
(2) Continuing medical education (CME): A continuing education session will be developed, featuring a presentation highlighting the relevance of heat-PIMs and providing guidance on appropriate adaptations for clinical practice.
(3) Open-access CME article: An open-access CME article will be created and published, detailing the content and practical application of the CALOR list.

These educational materials will be incorporated into the project partners’ teaching programmes. Broader dissemination will be supported through the project advisory board, institutions involved in the Delphi process and additional communication channels.

## 4. Conclusions

To our knowledge, this study protocol presents the first study attempting to collate a comprehensive list of recommendations for drug safety management during hot days and heatwaves. The multi-method protocol will allow for the development of relevant and actionable recommendations for a broad range of healthcare professionals.

## Data Availability

No new data were created or analysed in this study.

## Abbreviations

The following abbreviations are used in this manuscript:

heat-PIMs	Potentially Inadequate Medications during heat events
SHI	statutory health insurance
CoRe-Dat	CoRe-Net database
DWD	Deutscher Wetterdienst—German Weather Service
